# Prevalence of low back pain among elite Australian senior rowers

**DOI:** 10.1186/2052-1847-7-S1-O20

**Published:** 2015-08-11

**Authors:** Kellie Wilkie, Larissa Trease, Miranda Wallis

**Affiliations:** 1 Rowing Australia, Canberra, 2600, Australia; Bodysystem Physio, Hobart, 7000, Australia; 2 Rowing Australia, Canberra, 2600, Australia; Victorian Institute of Sport, Albert Park 3206, Australia; 3 Australian Institute of Sport, Canberra, 2600, Australia

## Background

Research shows that the prevalence of lower back (LBP) pain is high among rowers [1,2]. Previous methods of data collection on LBP in the Australia Rowing Team (ART) do not allow for comparison against other rowing populations. The purpose of this study was to collect the current prevalence of LBP across senior elite Australian rowers at one point in time, using methods comparable to those presently reported in the literature both in the general and sporting populations. A secondary aim of the study was to determine whether gender, type of rowing or weight category made a rower more susceptible to low back pain at a specific point in time.

## Methods

This was a cohort study, based on a cross-sectional survey of the 77 rowers selected as part of the 2014 Australian Senior Rowing Team. Participants were asked specific questions relating to their experience of LBP, particularly current, recent and lifetime LBP.

## Results

83% of the rowers surveyed had experience LBP at some point in their lifetime, whilst 57% had experienced LBP during their rowing career. 18% had experienced LBP within the past month of being surveyed and 8% had experienced LBP within the 24hour period before participating in the survey. A significant difference between sweep and sculling showed that sweep rowers were more likely to have experienced LBP than scullers in the 24 hours prior to the study. This was not evident when analysing for previous month, rowing lifetime or lifetime LBP prevalence. Gender and weight category did not make a rower more susceptible to LBP. These results are comparable to the experience of both other high-level rowing programs and the general population [1-3].

## Conclusion

The prevalence of LBP among senior elite Australian rowers is comparable or lower than that described previously among other rowing populations [1,2,4]. Elite Australian rowers have a lower rate of point and period LBP prevalence than the general population, but a higher rate of LBP over their lifetime [3]. Gender or weight category did not make a rower more susceptible to low back pain at a specific point in time. Sweep oar rowing made a rower more susceptible to LBP than sculling over a 24 hour period but this was not the case over in a one month prevalence period, suggesting other factors may be responsible.
